# Ancient dog mitogenomes support the dual dispersal of dogs and agriculture into South America

**DOI:** 10.1098/rspb.2024.2443

**Published:** 2025-06-18

**Authors:** Aurélie Manin, Regis Debruyne, Audrey Lin, Ophélie Lebrasseur, Evangelos Antonios Dimopoulos, Lucio González Venanzi, Sophy Charlton, Lachie Scarsbrook, Andrew Hogan, Anna Linderholm, Adam R. Boyko, Pauline Joncour, Mónica Berón, Paola González, Juan Carlos Castro, Silvia Cornero, Gabriel Cantarutti, Patricio López Mendoza, Ismael Martínez, Velia Mendoza España, Daniel Pavlovic, Luciano Prates, Francisco Prevosti, José Rogan, Adrián Oyaneder, Ximena Power, Susan deFrance, Michael Wylde, Belkys Gutierrez, Sandrine Grouard, Carolyn Freiwald, Jaime J. Awe, Claire E. Ebert, Julie A. Hoggarth, Juan Carlos Equihua, Grégory Pereira, Heidi Parker, Christine Lefèvre, Nicolas Goepfert, Elaine Ostrander, Greger Larson, Laurent Frantz, Christophe Hitte, Morgane Ollivier

**Affiliations:** ^1^ Palaeogenomics and Bio-Archaeology Research Network, School of Archaeology, University of Oxford, Oxford, UK; ^2^ Université Paris 1 Panthéon Sorbone, UMR 8096 ArchAm, CNRS, Paris, Île-de-France, France; ^3^ CBMN, UMR 5248, Plateforme Protéome, Université de Bordeaux, Pessac, Nouvelle Aquitaine, France; ^4^ UMR 7209 BioArch, Muséum national d'Histoire naturelle, Paris, Île-de-France, France; ^5^ Department of Anthropology, Smithsonian National Museum of Natural History, Washington, DC, USA; ^6^ Richard Gilder Graduate School, American Museum of Natural History, New York, NY, USA; ^7^ Centre de Recherche sur la Biodiversité et l'Environnement (CRBE), UMR5300, Université de Toulouse, Toulouse INP, CNRS, IRD, Toulouse, France; ^8^ Department of Veterinary Medicine, University of Cambridge, Cambridge, UK; ^9^ División Arqueología, Facultad de Ciencias Naturales y Museo, CONICET, Universidad Nacional de la Plata, La Plata, Argentina; ^10^ Department of Archaeology, University of York, York, Yorkshire, UK; ^11^ Animal Paleogenomics, Faculty of Veterinary Medicine, Ludwig-Maximilians-Universität, München, Germany; ^12^ National Human Genome Research Institute, National Institutes of Health, Bethesda, MD, USA; ^13^ Centre for Palaeogenetics, Department of Geological Sciences, Stockholm University, Stockholm, Sweden; ^14^ College of Veterinary Medicine, Cornell University, Ithaca, NY, USA; ^15^ CNRS, ECOBIO, UMR 6553, Université de Rennes, Rennes, Bretagne, France; ^16^ IDECU, Consejo Nacional de Investigaciones Científicas y Técnicas (CONICET), Universidad de Buenos Aires, Buenos Aires, Argentina; ^17^ Sociedad Chilena de Arqueología, Santiago, Chile; ^18^ Museo de Ciencias Naturales y Antropológicas Profesor Antonio Serrano, Paraná, Argentina; ^19^ Museo Universitario, FCEIA, Universidad Nacional de Rosario, Rosario, Argentina; ^20^ Museo de Historia Natural y Cultural del Desierto de Atacama, Santiago de Chile, Chile; ^21^ Centro de Estudios de Historia y Arqueología, Instituto de la Patagonia, Universidad de Magallanes, Punta Arenas, Chile; ^22^ Laboratorio de Zooarqueología, Carrera de Arqueología, Universidad Mayor de San Andrés, La Paz, Bolivia; ^23^ CIEM Aconcagua, San Felipe, Chile; ^24^ Museo de Ciencias Antropológicas y Naturales, Universidad Nacional de La Rioja (UNLaR), CRILAR-CONICET, La Rioja, Argentina; ^25^ Independent Researcher, Santiago de Chile, Chile; ^26^ Archaeology and History Department, University of Exeter, Exeter, UK; ^27^ Instituto de Alta Investigación (IAI), Universidad de Tarapacá, Arica, Chile; ^28^ Department of Anthropology, University of Florida, Florida Museum of Natural History, Gainesville, FL, USA; ^29^ Department of Anthropology, University of Florida, Gainesville, FL, USA; ^30^ BGL Arqueología EIRL, Trujillo, Peru; ^31^ Department of Sociology and Anthropology, University of Mississippi, Oxford, MS, USA; ^32^ Department of Anthropology, Northern Arizona University, Flagstaff, AZ, USA; ^33^ Department of Anthropology, University of Pittsburgh, Pittsburgh, PA, USA; ^34^ Department of Anthropology, Baylor University, Waco, TX, USA; ^35^ Instituto Nacional de Antropología e Historia, Ciudad de Mexico, Mexico; ^36^ CNRS / Institute Genetics and Development, Université de Rennes, Rennes, Bretagne, France

**Keywords:** palaeogenomics, phylogeography, animal domestication, migration, archaeology, Americas

## Abstract

Archaeological and palaeogenomic data show that dogs were the only domestic animals introduced during the early peopling of the Americas. Hunter–gatherer groups spread quickly towards the south of the continent, but it is unclear when dogs reached Central and South America. To address this issue, we generated and analysed 70 complete mitochondrial genomes from archaeological and modern dogs ranging from Central Mexico to Central Chile and Argentina, revealing the dynamics of dog populations. Our results demonstrate that pre-contact Central and South American dogs are all assigned to a specific clade that diverged after dogs entered North America. Specifically, the divergence time between North, Central and South American dog clades is consistent with the spread of agriculture and the adoption of maize in South America between 7000 and 5000 years ago. An isolation-by-distance best characterizes how dogs expanded into South America. We identify the arrival of new lineages of dogs in post-contact South America, likely of European origin, and their legacy in modern village dogs. Interestingly, the pre-contact Mesoamerican maternal origin of the Chihuahua has persisted in some modern individuals.

## 1. Introduction

Dogs (*Canis familiaris*) accompanied early waves of people who entered North America at least 15 000−16 000 years before present (BP) [1–3]. They were the only domestic animal introduced from Eurasia into the Americas prior to the arrival of European settlers. Archaeological and morphological evidence suggest that Arctic dogs were used for sledding, which would have been instrumental for human groups crossing from the cold tundra of Siberia [4–7]. Ancient DNA analyses have shown that all dogs preceding contact with European settlers (hereafter referred to as pre-contact dogs) possessed mitochondrial haplotypes belonging to the mitochondrial A2b clade that is specific to the Americas [2,3,8]. Dogs belonging to the A2b clade spread throughout the Americas, except in the Amazon basin where linguistic data suggest that they were unknown until the Europeans arrived, during the sixteenth century [9,10].

Archaeological data show that by 14 000 BP people had spread into Central and South America, likely following coastlines and riverways [11–13]. The distribution of human mitochondrial lineages in North, Central and South America suggests a rapid and single expansion [14,15], although nuclear DNA indicates a more complex pattern involving macro-regional migrations throughout the Holocene [16,17]. In contrast, archaeological evidence for dogs suggest that they were introduced much later in Central Mexico and further south. The earliest commonly accepted dog remains in Mexico, Ecuador and Peru are dated to 5200−5000 BP, suggesting that dogs only became widespread in these regions following the adoption of agriculture *ca* 7000 BP [18–21], but the reasons for their delayed dispersal in the region remain contentious [9,22,23].

However, identifying early dogs in American archaeological contexts has been complicated by the large diversity of canids that possess similar morphological features to dogs [24]. For instance, at Coxcatlan Cave, where the earliest dogs in Mexico have been identified, Flannery [18] reported the presence of coyote (*Canis latrans*), grey fox (*Urocyon cinereoargenteus*) and a large, likely extinct, fox-like canid species pre-dating 5000 BP. In fact, the largest diversity of extant wild canids [25] as well as extinct large foxes, such as *Dusicyon avus* [26], is found in South America. The limited number of features that can be used to morphologically distinguish extinct and extant species, and the high prevalence of fragmented bone remains in the archaeological record have resulted in many misidentifications [24,27,28]. On several occasions, due to the lack of observable diagnostic criteria or accurate comparative collections, definitive species identification between dog, coyote [29,30], wolf [3] or fox [31] has only been possible using mitochondrial DNA. However, understanding further structuring in ancient American dog populations has been limited by the low-resolution power of the mitochondrial control region that was used in previous studies [32–35].

Moreover, modern domestic dogs cannot be used as a genetic reference to address the delayed dispersal of dogs into Central and South America. The European colonization of the Americas led to the introduction of Eurasian dogs whose ancestry became the dominant feature of dog populations in the Americas today [8,32,36,37]. The indigenous American A2b mitochondrial clade, and its associated nuclear ancestry, has now almost vanished from the continent. Pre-contact maternal lineages have only been found in two modern American dogs so far: a village dog from Nicaragua and a Chihuahua from the United States [38,39]. Evidence from nuclear DNA indicates that the proportion of Native American ancestry in modern American dogs (such as the Chihuahua and the Xoloitzcuintli) is approximately 3–4% at most [40].

To date, archaeogenomic studies of American dogs have largely focussed on North America [6,8] while ancient Central and South American dogs are poorly represented [31,41,42]. In order to address this gap in the research and estimate arrival time of dogs to these southern regions, as well as investigate dog population movements, we produced 62 new ancient mitochondrial genomes and eight modern mitochondrial genomes. Mitogenomes are uniparental markers that allow the reconstruction of the evolution of maternal lineages through time and space, and we used these markers here as a first evidence of dog population dynamics in Central and South America.

## 2. Material and methods

**Figure 1.**
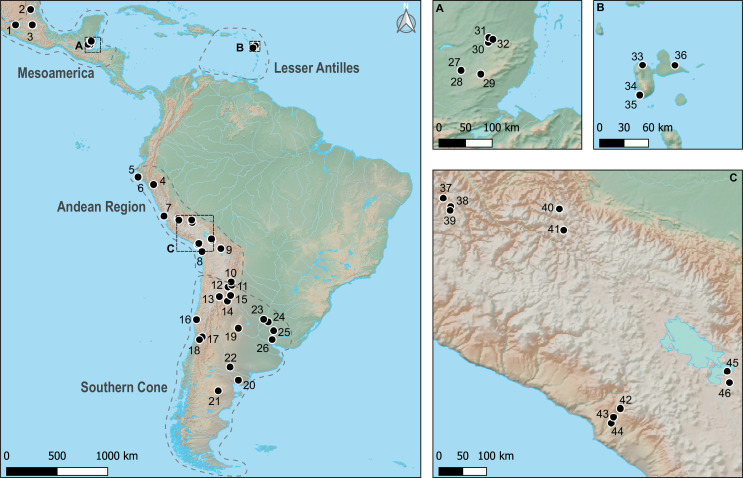
Distribution of archaeological samples analysed in this study. The number of individuals per site is indicated between brackets: 1 = Malpaís Prieto (*n* = 1); 2 = Vista Hermosa (*n* = 4); 3 = Tizayuca (*n* = 25); 4 = Chondorko (*n* = 1); 5 = Huaca Amarilla (*n* = 18); 6 = Huaca Grande (*n* = 9); 7 = Chilca (*n* = 2); 8 = Playa Miller 4 (*n* = 1); 9 = Iroco (*n* = 1); 10 = Pucará de Tilcara (*n* = 3); 11 = Finca Tolaba (*n* = 1); 12 = Santa Rosa de Tastil (*n* = 1); 13 = Antofagasta de la Sierra (*n* = 1); 14 = Loma Rica de Shiquimil (*n* = 1); 15 = Pampa Grande - Caverna III site (*n* = 1); 16 = El Olivar (*n* = 6); 17 = Los Nogales (*n* = 1); 18 = Talleres y Cocheras (*n* = 1); 19 = Observatorio Astronómico (*n* = 1); 20 = Angostura 1 (*n* = 1); 21 = Sierra Apas (*n* = 1); 22 = Chenque I (*n* = 1); 23 = La Lechuza (*n* = 1); 24 = Arroyo Las Mulas 1 = (*n* = 1); 25 = Lower Uruguay River (*n* = 1); 26 = Cerros de los Pampas (*n* = 1); 27 = Zacpeten (*n* = 2); 28 = Nixtun Ch'ich' (*n* = 6); 29 = Ucanal (*n* = 3); 30 = Xunantunich (*n* = 2); 31 = Cahal Pech (*n* = 10); 32 = Baking Pot (*n* = 2); 33 = Habitation La Ramée (*n* = 1); 34 = Cathédrale de Basse Terre (*n* = 1); 35 = Gare Maritime (*n* = 2); 36 = Morel (*n* = 5); 37 = Rosamachay (*n* = 1); 38 = Wari (*n* = 1); 39 = Wichqana (*n* = 1); 40 = Lares (*n* = 8); 41 = Cuzco (*n* = 4); 42 = Torata Alta (*n* = 1); 43 = Omo (*n* = 1); 44 = Río Muerto (*n* = 1); 45 = Qiwaya (*n* = 3); 46 = Tiwanaku (*n* = 2).

We extracted DNA from 131 archaeological canids and 12 modern dogs spanning the last 5000 years, whose location ranged from Central Mexico to northern Patagonia (figure 1, electronic supplementary material, table S1). Contextual information on each archaeological site is provided in electronic supplementary material, text 1. Overall, the samples can be divided between four broad cultural areas: (i) Mesoamerica, a region extending from central Mexico to Western Honduras and Nicaragua; (ii) the Lesser Antilles; (iii) the Andean region that ranges along the Pacific coast of South America and on the western slope of the Andes across Ecuador, Peru, Bolivia and Chile; and (iv) the southern cone that gathers the eastern slope of the Andes, the Argentinian and Uruguayan pampas and Patagonia. This sampling has largely been driven by the availability of well-contextualized dog remains, and the apparent lack of individuals in the tropical lowlands reflects both the possible absence of pre-contact dogs in many of these regions [22] and poor bone preservation [43].

Eight samples found on four sites of the Lesser Antilles were tested with PCR assays without conclusive results and they were not used in downstream analyses. We performed shotgun sequencing of 123 ancient individuals from 43 archaeological sites as well as on the 12 modern dogs (figure 1, electronic supplementary material, table S1, text 1) using paired-end Illumina technologies (NextSeq and HiSeq, electronic supplementary material, text 2).

Following shotgun screening, which was used to estimate the amount of endogenous DNA in each sample, we employed a combination of mitochondrial in-solution hybridization capture and deep shotgun sequencing to generate 35 high-coverage (>10×), seven medium-coverage (5−10×) and 28 low-coverage (<5× but > 50% of the genome covered) mitochondrial genomes from timeframes spanning 1700 years (electronic supplementary material, table S1). These were analysed alongside publicly available ancient and modern canid sequences (electronic supplementary material, tables S2–S4).

Species identification was genetically investigated and confirmed based on a competitive mapping of the reads with available nuclear and mitochondrial genomes from canid species present in the Americas using HAYSTAC [44] (electronic supplementary material, text 2, §1.4.3). A maximum likelihood (ML) tree was built from medium and high-coverage mitogenomes using IQ-Tree [45] (electronic supplementary material, text 2, §1.4.4). The phylogenetic position of the low-coverage mitogenomes was then assessed using this ML tree as a constrained tree (electronic supplementary material, text 2, §1.4.5). Nucleotide and haplotype diversity indices were computed on the high-coverage mitogenomes using DNAsp v. 6 [46], and Arlequin v. 3.5 [47] was used to perform analysis of molecular variance (AMOVA). All statistical and graphical representations were computed with R v. 4.0.2 [48]. Bayesian-inferred phylogenies were built from medium- and high-coverage mitogenomes using BEAST2 v. 2.6.3 [49]. For this specific analysis, we created three different datasets, each consisting in 218 mitochondrial genomes that resulted from the combination of a fixed set of sequences and a variable set of modern sequences randomly selected among publicly available sequences. These datasets were analysed in parallel to assess the robustness of the results to potential sample bias effects [3]. The fixed set in each dataset consisted of 164 sequences comprising 148 high-coverage archaeological dog mitochondrial genomes from the Americas and Eurasia and 16 modern dog mitochondrial genomes from American dogs and American breeds. The variable set consisted of 54 modern dog mitochondrial genomes from Eurasia and Africa (electronic supplementary material, table S4). The random selection of modern genomes was repeated three times to create the three different datasets of 218 sequences. For each of the three datasets, we tested both strict and relaxed molecular clock models. We compared the clock models using nested sampling [50] and retained the relaxed clock model that gave a higher likelihood (electronic supplementary material, table S5). To further test the demographic history of dogs in the Americas, we produced a coalescent Bayesian skyline analysis on all the 106 ancient and modern American dog mitogenomes that were available (electronic supplementary material, table S6 and text 2, §1.4.7).

## 3. Results and discussion

### (a) Identifying Mesoamerican and South American dogs

The samples analysed in this paper were initially selected due to their morphological and morphometrical attribution to *Canis familiaris* based on previous publications using geometrics morphometrics [51] and morphoscopic criteria [52,53] (see details in electronic supplementary material, text 2). Of the 123 ancient individuals that were shotgun sequenced, competitive mapping of the generated reads using HAYSTAC [44] confidently identified 120 dogs while three samples could not be identified due to low DNA contents. Noteworthy, we genetically confirmed the identification of the earliest dog from the Southern Cone (La Lechuza, 2691−2185 cal. BP, [52]). The high correspondence between morphological and molecular identification validates the morphological criteria that were used in previous zooarchaeological studies. Our result is particularly important in South America where different species of dog-size canids coexisted, such as *Chrysocyon brachyurus, Lycalopex culpaeus* and the extinct *Dusicyon avus* [21,53–56].

### (b) Assessing Mesoamerican and South American dog population diversity

Following the introduction of dogs into the Americas, the A2b mitochondrial clade radiated into four sub-haplogroups: A2b1, A2b2, A2b3 and A2b4 [6,8]. All four clades have been described in North America [3,6,8,42]. In addition, nine individuals from Central and South America have been previously sequenced, all of which belonging to the A2b1 clade [8,31,41,42]. Using an ML approach, we constructed a phylogenetic tree based on 42 newly produced mitochondrial genomes (37 archaeological samples and five modern samples) with over 5× coverage that we compared to 218 published sequences (electronic supplementary material, figure S1 and table S3). The phylogenetic position of 28 low-coverage samples was then assigned based on this tree (electronic supplementary material, table S7).

Thirty-six archaeological individuals fell into the previously described A2b1 sub-haplogroup, forming a monophyletic clade with the previously sequenced Central and South American dogs. Twenty-three archaeological individuals with low-coverage genomes were further assigned to the A2b1 haplogroup. None of the samples analysed in this study belonged to another of the A2b sub-haplogroups, showing that A2b1 was the only one that radiated toward the south of the continent.

One archaeological dog from Torata Alta, found in a colonial context (electronic supplementary material, text 1), fell in haplogroup C, far from the A2b clade that characterizes the pre-contact American dogs. In addition, two archaeological individuals with low mitochondrial coverage, from Tiwanaku and El Olivar, respectively, were assigned outside of the A2b clade. The dogs were not directly dated but their mitochondrial haplogroup strongly suggests they were post-contact animals. The intrusion of recent material into archaeological contexts has been observed previously in Bolivian dogs [31] as well as numerous other taxa in Europe, such as chickens [57] or reindeer [58], among others (see further discussion in electronic supplementary material, text 2, §2.4). Finally, all eight modern dogs from Lares and Cuzco, in the Central Andes, fell outside the A2b clade (belonging to the A, B and C haplogroups, electronic supplementary material, figures S1 and table S7). These 11 samples support previously published results obtained on village dogs [38] to illustrate the rapid and almost complete post-contact replacement of ancient American dogs.

### (c) The dispersal of pre-contact dogs into Central and South America

**Figure 2.**
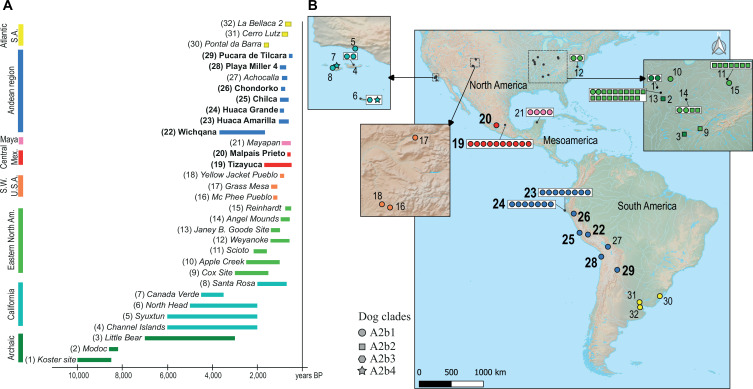
Mitochondrial diversity of the pre-contact American dogs (see electronic supplementary material, table S3 for the list of samples). A = chronology of the sites considered in this study; sites whose samples are published here are indicated in bold; the numbers refer to the sites location on the following map. B = geographical distribution of the samples in the different sites; the larger font size refers to the new sites analysed here.

To better understand the pattern of dog dispersal into South America, we analysed the distribution of pre-contact dog populations in North America, Mesoamerica and South America using the 31 high-quality, pre-contact, newly generated mitochondrial genomes that we compared to 55 previously published mitochondrial genomes from the Americas [8,31,41,42] (electronic supplementary material, tables S1, S8, S9; figure 2A). We focussed our analysis on the population dynamics of the maternal lineages with the postulate that, at this stage, there is no evidence for a sex-biased behaviour in the dispersal of dogs.

We divided the genomes between North America, Mesoamerica and South America, based on their cultural and geographic origin (figure 2B). The phylogeny shows a clear divide between dogs from North America, Mesoamerica and South America (electronic supplementary material, figure S1). We then calculated the pairwise distance between each mitogenome, and our results indicate a greater proximity between individuals from the same geographic region than between regions (electronic supplementary material, figure S2). Interestingly, the Mesoamerican individuals appear closer to the South American dogs than to the North American individuals, which might be explained by a rapid dispersal towards the south of the continent. In addition, most of the genomes that were available for the study are concentrated in the last 2000 years (figure 2A), and we cannot rule out the hypothesis that a ghost lineage from North America would slightly change this conformation by being closer to the Mesoamerican individuals (see further discussion in electronic supplementary material, text 2, §2.1).

We confirmed the proximity between the individuals from the same geographical region on a finer scale using the AMOVA. This analysis partitions the total genetic variance into covariance components based on the difference observed at the various levels of population hierarchy (among regional groups, among subpopulations within regional groups and within the total population). Hence, we grouped the specimens into seven geographical regions (Eastern USA, Southwestern USA, California, Central Mexico, Maya area, Andean region, South American Atlantic Coast), with the addition of an eighth group for Archaic samples from Eastern USA, as they represent a subset that predates all other samples from the same region (figure 2A,B). Within each group, the samples were split by site (figure 2A; electronic supplementary material, text 2). This analysis revealed that most variation was found between groups (*V*_a_ = 44.91%; *p*‐value < 0.00001), hence between geographical regions, rather than within regions (*V*_b_ = 16.64%; *p*‐value = 0.00098) or within archaeological sites (*V*_c_ = 38.66%; *p*‐value < 0.00001). Our result differs from modern domestic populations, where repeated translocations tend to diminish the patterns of phylogeographic structuring that are frequently observed in wild populations (i.e. [59,60]; further discussion in electronic supplementary material, text 2*,* §2.2). Even though the sample size was small, this trend suggests limited movement of female dogs between regions of the Americas in pre-contact times.

We further tested the isolation-by-distance (IBD) dispersal model between each dog using a Mantel test between pairwise genetic distance and geodesic distance. The results were significant (*r* = 0.4441; *p*‐value = 1 × 10^−4^) and correlation increased when using the least-cost distance through landmasses (*r* = 0.4784; R package geodist). As dogs arrived via North America, our result suggests that they followed a one-way dispersal through the Americas, entering Central America and, subsequently South America. The population appears largely structured and, although cultural exchanges and population movements existed between the different regions of the Americas [61], dogs likely did not take part in these exchanges.

Moreover, diversity indices for each archaeological site or population are generally higher in North America than in Central and South America, further supporting a stepping-stone model of dispersal [62] and repeated bottlenecking that resulted as dogs spread south (electronic supplementary material, table S10). Yet, a later population increase and subsequent diversification likely occurred in each of the regions, as suggested by the relatively high genetic diversity observed in the site of Huaca Amarilla, on the northern coast of Peru.

The use of mitochondrial DNA conceals part of the genomic diversity and, at this stage, we cannot exclude that geneflow also occurred in a south-to-north direction. The development of nuclear DNA analyses in cattle, for instance, revealed a male-orientated introgression of zebu in the Near-East *ca* 4000 BP [63]. Likewise, in the Americas, human nuclear DNA exhibits a different pattern of population structure from the mitogenome [16,17]. The inclusion of nuclear data would therefore provide a more complete overview of dog population dynamics and inform about male-specific movements and fine-scale regional exchanges.

### (d) Investigating the dual dispersal of dogs and maize agriculture in pre-contact America

Based on zooarchaeological evidence, domesticated dogs are hypothesized to have arrived in South America after—or during—the development of maize agriculture approximately 7000 BP [21,64,65]. Under this scenario and given the high degree of population structure between North and South America, we expect this time to be either younger or coincide with the time to the most recent common ancestor (TMRCA) between haplogroups found exclusively in the north and in the south. We tested this hypothesis using the time calibrated phylogeny built with BEAST2 [49].

**Figure 3.**
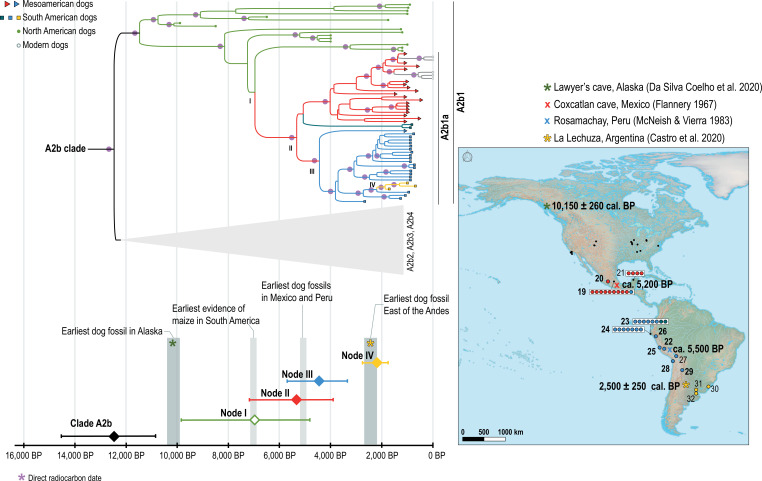
Time divergence estimates (bottom) for the nodes highlighted on the time-modelled Bayesian tree (top; relaxed clock, dataset 1). Posterior probabilities higher than 80% are represented by a pink circle on the tree and a filled diamond on the chronology. Empty diamonds indicate a posterior probability lower than 80%. Location of the radiocarbon and stratigraphically dated earliest fossils for North America, Mesoamerica, the Andean region and Southern South America are indicated on the map, along with the sites where dog DNA has been retrieved (each circle represent an individual). Mesoamerican and South American site names are given in figure 2A.

The time-modelled phylogeny was congruent with the ML topology obtained from IQ-Tree and the three datasets tested in parallel resulted in similar tree topologies (electronic supplementary material, figures S3–S5, table S11). All 36 newly produced, high-quality, mitochondrial genomes from pre-contact dogs form a monophyletic lineage within the A2b1 clade (hereafter A2b1a, figure 3), along with previously published dog mitogenomes from Central and South America. Interestingly, the A2b1a clade consisted solely of Mesoamerican and South American dogs and does not include previously published ancient dog mitogenomes from Canada and continental USA. This indicates that the dogs that were brought into the region originated from a genetically (at least mitochondrially) homogeneous population. One clade, gathering two South American samples (Nunura_8 and Nunura_15), had a varying position depending on the dataset used for the analysis, but always included into the A2b1a clade. The position of this clade (dark blue, figure 3) was never statistically supported, and the implication of this result is further discussed in electronic supplementary material, text 2*,* §2.3. The nested sampling identified dataset 1 as being best supported by the model (electronic supplementary material, table S5) and therefore all the results discussed below arise from this dataset. The divergence ages and posterior probabilities for each of the nodes were nevertheless highly similar (electronic supplementary material, table S11), which supports the validity of the model presented here.

The divergence age of clade A2b was 12 473 BP (95% highest posterior density [HPD]: 14 553–10 861), which is congruent with previous estimates [2,3]. TMRCA between North American dogs, and Mesoamerican and South American dogs (node I, figure 3), was 6962 BP (95% HPD: 9845–4810; electronic supplementary material, table S11). This is more recent than the introduction of dogs to the Americas and confirms that the common ancestor of these haplogroups was an American dog. The analysis of demographic history of the American dogs indicates that their population grew progressively through time (electronic supplementary material, figure S6 and text 2, §2.4).

Subsequent splits (nodes II and III, figure 3) between Mesoamerican and South American dogs and among northern South American dogs largely overlap but follow a general north-to-south diversification gradient (figure 3). We estimated the coalescence time of all Mesoamerican and South American dogs to 5329 BP (node II, 95% HPD: 7174–3894; electronic supplementary material, table S11). Although this does not represent a population split, given that all Mesoamerican and South American pre-contact dogs belong to this clade, this timing provides a reliable upper bound for the time by which dogs were introduced from North America. This timing largely postdates the first peopling of South America, approximately 15 000 BP [8], and supports a later introduction of dogs.

Multiple studies of animal and plant domestication show that molecular clocks tend to provide earlier dates than archaeological evidence, which can be considered as the maximum age and minimum age for a given event, respectively (e.g. [66–68]). With the earliest archaeological evidence of dogs in central Mexico and northern South America dating from 5200 to 5000 BP [18,19,24], we can place the arrival of dogs in the region between approximately 7200 BP and 5000 BP. This timing coincides with the early introduction of maize from Mexico to South America approximately 7000 BP [64]. During the next two millennia, maize agriculture consolidated. At least one other wave of introduction of maize in South America has been identified, as well as its backward expansion to Central America, likely by 4700 BP [69], supporting the existence of recurrent exchanges between the regions. Studies of uniparental markers in human populations have also highlighted the expansion of Y chromosome haplotype Q1a3a approximately 7000–5000 years BP [70,71]: a haplotype common to Mexico, the northern Amazon and the Andes, that has been connected to the expansion of agricultural practices. Together, these three lines of evidence converge on the movement of people and dogs in association with the spread of agriculture rather than supporting the hypothesis that dogs were introduced during the initial peopling of South America by hunter–gatherers. This dispersal happened in a context of early agrarian societies and frequent human mobility throughout the macro-region. The study of ancient human nuclear genomes from the Andean region indicates that mobility largely decreased after 5000 BP with the emergence of economic and political differentiations [72].

The split between Andean dogs and dogs from the Atlantic coast (node IV) occurred much later. TMRCA is estimated to 2193 BP (95% HPD: 2766–1756 BP; electronic supplementary material, table S11), which is consistent with the direct radiocarbon dating of the earliest fossil found east of the Andes [52] but also with a subsistence shift towards the adoption of maize agriculture [73] (see further discussion in electronic supplementary material, text 2*,* §2.3). Finally, and although agriculture was probably the main support for the southern spread of dogs, they were later adopted by societies characterized by a large range of economies, from horticulturists to nomadic hunter–gatherers, from southeastern Brazil to Patagonia [21,41,55,74,75]. Archaeological evidence, and particularly some exceptional mortuary deposits, demonstrates that, in these societies, human and dogs were bound by close ties [55,74,75].

### (e) The post-contact population replacement of American dogs and the ancient American genetic legacy of the Chihuahua breed

To better understand the population replacement that occurred in Mesoamerica and South America following the European colonization, we examined the eight newly generated mitochondrial genomes from modern dogs from the Lares and Cuzco valleys (Peru) and the three post-contact archaeological dog mitogenomes from El Olivar (Chile), Tiwanaku-Kalasasaya (Bolivia) and Torata Alta (Peru). We analysed them along published data to explore the post-contact dynamics of American dogs’ maternal lineages (electronic supplementary material, figures S1, S7 and table S7). The dog from Torata Alta and two dogs from Lares (OL1517 and OL1518) belong to clade C and cluster with ancient European dogs dating to 4000−6000 cal. BP. This clade was already present in Europe 14 000 to 15 000 years ago [42,76]. Three additional dogs from Lares (OL1516) and Cuzco (OL1522 and OL1524) are scattered throughout clade B and group with a post-contact sample previously identified in Bolivia (CAN001 [31]), that diverged from other modern European breeds less than 2000 BP (electronic supplementary material, figures S3–S5). Finally, the dog from El Olivar, the dog from Tiwanaku-Kalasasaya, two dogs from Lares (OL1515 and OL1514), and one dog from Cuzco (OL1525) belong to clade A, although they are outside of clade A2b. They are scattered among modern breeds, but we note the close proximity between the dog from Tiwanaku-Kalasasaya and a previously published Bolivian sample (CAN002) from Chiripa dated from the seventeenth to twentieth century [31] (electronic supplementary material, figure S1). Given the long history of these three clades in Eurasia [40,77–79] their identification in South American sites provide evidence of the introduction of new dog clades to the Americas at the time of the European contact.

The fact that some dogs found in the Lares valley today cluster with a sixteenth century dog from Torata Alta (AL2839), and a dog previously found in Bolivia (CAN001 [31]) highlights a local continuity in the Central Andes. The dogs from Lares are likely descendants of dogs introduced into the region up to 500 years ago. In the same way, the DNA similarity we observed between the dog from Kalasasaya-Tiwanaku and a previously published, historical, individual from Chiripa is another evidence of local continuity. In contrast, we did not observe survival of the A2b clade in the modern samples analysed from Peru. Colonial rules and stray dog population control, as implemented under the Spanish crown [80], may have quickly led to the replacement of the indigenous dogs.

Interestingly, one modern Chihuahua dog from the USA included in our reference panel still carries mitochondrial DNA from the A2b clade and is closely associated with Mesoamerican dogs (electronic supplementary material, figures S1, S3–S5). The TMRCA between the modern Chihuahua and the closest archaeological dog (Az−1935) is 1520 BP (95% HPD: 1949–1187 BP) that predates the arrival of the Europeans and supports its American origin. A previous genomic analysis showed that pre-contact ancestry was still present in low proportions in the nuclear genome of the Chihuahua (<5% [40]). Here, we show that the ancient American origin of this lineage, still preserved in the non-recombining mitochondrial DNA, can be more precisely traced to Mesoamerican dogs and agrees with the historical origin of the breed. We found the earliest mention of the Chihuahua dog in a 1862-written correspondence between the director of the Acclimatation Society of Paris and the French Naval Minister aiming at importing this Mexican dog for the 1863 World Exhibition of dog breeds [81]. Later descriptions from 1868 indicate that the Chihuahua was already a small dog, reaching only 14 cm at the shoulder [82].

Although Mexican hairless dogs were also showcased at the 1863 World Exhibition [83], the breed nearly vanished at the beginning of the twentieth century and was only saved by the Mexican Kennel Club in the 1940s from a few Mexican dogs (World Kennel Club, FCI-St. no. 234). Repeated admixture with unrelated breeds (e.g. German shepherd) has completely transformed the genetic make-up of the breed [84].

## 4. Conclusion

Dog dispersal into South America appears to be associated with the expansion of agricultural practices around 7000 to 5500 years ago, following a stepping-stone dispersal model throughout the continental landmasses. This long delay is surprising, as human populations were already present in South America for at least 8000 years prior to the arrival of dogs and different hypotheses can be proposed. The earliest fisher–hunter–gatherers that inhabited central America [85] and reached South America [86] may have not found many subsistence advantages from keeping dogs [9]. Moreover, crossing the Central American land bridge required the dogs to adapt to a tropical environment characterized by many new diseases, insects and parasites [22], and they may have been easy prey for local predators [9]. These risks were actually recorded in some early European chronicles from South America, indicating that they were still important factors reducing the survival of dogs during the eighteenth century [87]. Under this scenario, only few dogs may have reached South America during its initial peopling, albeit it is important to note that, to date, no dog remains have been confidently identified in these early settlements. According to the data produced in this paper, this (still hypothetical) early population would have been replaced by the later arrival of dogs accompanying the development of agrarian societies. In Europe, the in-depth analysis of Epipalaeolithic and Neolithic dogs remains have shown that the early domestic dogs have been replaced by the arrival of animals from the Levant during the Neolithic transition, between 9000 and 6000 BP [40,79] and a similar scenario could have occurred in the Americas. Only the extended analysis of Archaic sites as well as the addition of nuclear genomic data can resolve this controversy.

The Mesoamerican and South American early agrarian communities, however, may have found more uses for dog keeping and breeding, whether it was a source of meat [88] or for protection and companionship. The production of food surpluses may have also encouraged dog scavenging and allowed them to proliferate in the vicinity of the agrarian settlements. Dog dispersal towards the south may have been human-mediated but we cannot exclude the possibility that some feral dogs may have also spread on their own and relocated based on available resources, kin competition for mates or inbreeding avoidance [89]. The systematic addition of isotope analyses to the study of early individuals would refine our understanding of human–dog proximities and help define the modalities of their dispersal.

From the sixteenth century onwards, the introduction of dogs by the European settlers impacted the population of pre-contact dogs, whose genomic signature is now almost completely diluted in European dogs ancestry. One notable exception are Chihuahuas that still carry, for some individuals, a pre-contact maternal line, providing evidence of their Mexican origin.

This study sheds new light on the depth of the shared histories of human societies and dogs in the Americas and highlights the importance of agrarian societies in the spread of dogs. While dog domestication is intrinsically associated with hunter–gatherer groups [76,78], there is a growing body of evidence that early farmers were instrumental for dog dispersal. A similar pattern has been found in East Asia, where dog population expansion around 7500 BP has been associated with the spread of agricultural communities across the region and a growth in food supply [90].

Further investigations on extended collections will also be necessary to better understand the role of environmental barriers in the dispersal of dog in the Americas as well as the regional trends of their breeding, selection and exchanges. In-depth analyses of nuclear genomes coupled with the thorough study of dog social and cultural conception through time have the power to inform more precisely on the demographic history of the past populations (e.g. [37]) and would bring further insights into the dynamics of dog population history in the Americas.

## Data Availability

All the newly produced mitogenomes used in the analyses have been deposited in Dryad [91]. Supplementary material is available online [92].
